# Brennpunkt: psychiatrischer Maßregelvollzug und Lösungsansätze

**DOI:** 10.1007/s00115-025-01854-2

**Published:** 2025-07-09

**Authors:** Jürgen L. Müller, Matthias Koller

**Affiliations:** 1 Schwerpunktprofessur für forensische Psychiatrie, Asklepiosklinik für Forensischer Psychiatrie, Universitätsmedizin Göttingen, Rosdorfer Weg 70, 37081 Göttingen, Deutschland; 2 Göttingen, Deutschland

## Problemskizze

Die aktuelle Situation im psychiatrischen Maßregelvollzug ist besorgniserregend. Schon eine 2021 von der Deutschen Gesellschaft für Psychiatrie und Psychotherapie, Psychosomatik und Nervenheilkunde (DGPPN) unter den Maßregelvollzugseinrichtungen in Deutschland durchgeführte Umfrage [10] zeigte einen erheblichen Überbelegungsdruck mit der Not zur Unterbringung in Mehrbettzimmern oder gar in ehemaligen Therapie- oder Gemeinschaftsräumen. Die Überbelegung führte zu verbalen und körperlichen Übergriffen zum Nachteil von Mitpatienten und Behandelnden. Notwendige Therapien konnten häufig nicht stattfinden.

Seither hat sich die Situation nicht zum Besseren verändert. Zwar zeichnet sich mittlerweile eine deutliche Entlastung der Entziehungsanstalten durch die am 01.10.2023 in Kraft getretene Überarbeitung des Rechts der Unterbringung nach § 64 Strafgesetzbuch (StGB; Bundesgesetzblatt [BGBl.] 2023 I Nr. 203 vom 02.08.2023) ab. Gleichzeitig steigt aber die Zahl der Unterbringungen in einem psychiatrischen Krankenhaus nach § 63 StGB nachhaltig an. Die Strafverfolgungsstatistik des Statistischen Bundesamtes ([8], seit Jg. 2017) weist eine Zunahme der jährlichen Unterbringungsanordnungen nach dieser Vorschrift von 804 im Jahr 2017 auf 1202 im Jahr 2023 und damit um 50 % aus. Das heißt: 2023 wurden 400 Patienten mehr zugewiesen als 2017; auch für diese Patienten müssen dann für durchschnittlich 8 Jahre Plätze im Maßregelvollzug bereitgestellt und Behandlungsangebote gemacht werden. Ein 2016 unternommener Versuch des Gesetzgebers (Gesetz vom 08.07.2016 – BGBl. I 2016, Nr. 34 vom 14.07.2016, S. 1610), dem seit Jahren beobachteten Anstieg der Zahl der nach § 63 StGB untergebrachten Personen und der durchschnittlichen Unterbringungsdauer durch Schärfung der Anforderungen an die Verhältnismäßigkeit der Unterbringung entgegenzuwirken, hat jedenfalls eine deutliche Zunahme der jährlichen Zuweisungen also nicht verhindern können [7].

Zugleich haben sich die zugrunde liegenden Störungsbilder deutlich verändert. Zufolge der Strafverfolgungsstatistik wurde mehr als 80 % der neu nach § 63 StGB untergebrachten Personen eine aufgehobene Schuldfähigkeit bei Begehung ihrer Anlasstat(en) attestiert. Mitte der 1980er-Jahre lag dieser Anteil bei 56 % und 2011 bei 70 % ([8], seit Jg. 1986). Dies deutet darauf hin, dass die Anlasstaten zuletzt deutlich häufiger als früher unter dem Einfluss einer akuten Psychose begangen wurden. Dagegen wurden Patienten mit einer Störung der sexuellen Präferenz oder mit Persönlichkeitsstörungen nur noch selten zugewiesen.

Die Entwicklung der Zuweisungen korrespondiert mit den Befunden der jährlichen Erhebung des sog. Kerndatensatzes zur Belegung der Kliniken des Maßregelvollzuges [3]. Auch hier findet sich eine Zunahme an Patienten mit Schizophrenie von 2937 Patienten entsprechend 44 % im Jahr 2010 auf 3018 Patienten entsprechend 56 % im Jahr 2019.

Außerdem ist dem Kerndatensatz ein Anstieg der Zahl der einstweilig nach § 126a Strafprozessordnung (stopp) untergebrachten Patienten von 724 im Jahr 2010 auf 1020 im Jahr 2019 zu entnehmen. Extrapoliert man die Zahlen aus der Strafverfolgungsstatistik zur Schuldfähigkeit ist davon auszugehen, dass bei über 80 % eine psychotische Störung zugrunde lag. Dies entspricht auch den Daten zu den einstweiligen Unterbringungen im Berliner Maßregelvollzug [1].

Die Behandlung von Untergebrachten, bei denen im Urteil von Schuldunfähigkeit ausgegangen wurde, hat sich in der Vergangenheit als ausgesprochen erfolgreich erwiesen. Nach der bundesweiten Rückfalluntersuchung „Legalbewährung nach strafrechtlichen Sanktionen“ [6] wurden von insgesamt 354 im Jahre 2004 aus der isolierten Unterbringung nach § 63 StGB entlassenen und unter Führungsaufsicht gestellten Patienten in einem insgesamt 12-jährigen Beobachtungszeitraum bis 2016 nur 64 (entsprechend 18 %) rückfällig.

Dennoch beschreibt der Kerndatensatz eher noch einen Anstieg der durchschnittlichen Unterbringungsdauer bis zur Beendigung der Maßregel nach § 63 StGB: Lag sie im Jahre 2010 bei 2700 Tagen entsprechend 7,4 Jahre, stieg sie bis 2019 auf 3697 Tage entsprechend 10,1 Jahre an [3]. 2023 war sie dann wieder auf 2898 Tage (bundesweit ohne Baden-Württemberg) entsprechend 7,94 Jahre gesunken^1^ [4].

Dies wirft die Frage auf, warum bei weitgehender Konstanz der Prävalenz von Schizophrenie in der Bevölkerung die Betroffenen heute vermehrt nach § 126a StPO untergebracht werden und für eine lange Dauer in die Unterbringung nach § 63 StGB kommen und ob und was man dagegen tun sollte und kann.

## Herausforderungen

Der im Anschluss an das LWL(Landschaftsverband Westfalen-Lippe)-Expertengespräch „Psychiatrie und Recht“ 2023 in Münster formulierte „Weckruf“ [5] beschreibt die Versorgung schwer psychisch kranker Menschen als defizitär und benennt als Gründe dafür eine unzureichende Koordination, Steuerung und wechselseitige Abstimmung bestehender Versorgungs- und Finanzierungssysteme. Infolgedessen gebe es kaum längerfristige ambulante und aufsuchende und gegebenenfalls auch präventive Angebote für diese Personengruppe. Zudem sei der Zugang zu unterstützenden Wohnangeboten erschwert. Wenn ein Betroffener unter diesen Umständen krankheitsbedingt eine Straftat begeht, kann also die Unterbringung im Maßregelvollzug als alleiniger Ausweg verbleiben, weil es an den erforderlichen Ressourcen fehlt, um Krankheit und Gefährlichkeit mit ambulanten Maßnahmen zu begegnen. Dem entspricht es, wenn sich ausweislich einer Untersuchung in Berlin die Mehrzahl der nach § 126a StPO untergebrachten Patienten zuvor in allgemeinpsychiatrischer Behandlung befand, häufig aus der Obdachlosigkeit kam und keine kontinuierliche Behandlung wahrgenommen hatte [1]. Dass in Anbetracht der schwierigen Versorgungssituation zunehmend auch weniger schwerwiegende Taten zum Anlass für eine Unterbringung nach § 126a StPO und § 63 StGB genommen werden, ist bislang nicht hinreichend belegt.

Eine zusätzliche Herausforderung besteht in dem steigenden Anteil an Patienten mit Migrationshintergrund. Sprachliche Schwierigkeiten erschweren vielfach die Umsetzung therapeutischer Angebote. Lockerungen und rehabilitative Maßnahmen werden durch einen unsicheren Aufenthaltsstatus infrage gestellt, wenn nicht gar durch die fehlende Finanzierung und das beschränkte Angebot an geeigneten aufnahmebereiten Einrichtungen verunmöglicht. Ist es deshalb im Einzelfall zu einer krankheitsbedingten Straftat gekommen und eine Unterbringung im Maßregelvollzug angeordnet worden, sind therapeutische und rehabilitative Maßnahmen bei dieser Patientengruppe auch hier oftmals nur unter größten Schwierigkeiten umzusetzen. Sprachbarrieren erschweren die differenzierte Auseinandersetzung mit den straftatrelevanten Störungsbildern. Bei einem unsicheren Aufenthaltsstatus können Erprobungen erschwert und es kann sogar unmöglich werden, einen sozialen Empfangsraum zu entwickeln. Ohne erfolgreiche Lockerungserprobung und Integration in einen geeigneten sozialen Empfangsraum ist eine Entlassung aber kaum verantwortbar.

Angesichts des hohen Anteils psychotisch erkrankter Patienten an den nach § 63 StGB untergebrachten Personen dürfte sich auch die 2011 ausformulierte Rechtsprechung des Bundesverfassungsgerichts (BVerfG, [2]) auswirken, wonach eine medikamentöse Behandlung gegen den Willen nur unter engen Voraussetzungen in Betracht kommt. Zwar gibt es inzwischen bundes- und länderrechtliche Regelungen, die die medikamentöse Behandlung auch gegen den Willen des Betroffenen unter strikter Beachtung der strengen sachlichen und prozeduralen Vorgaben des BVerfG ermöglichen. Die in den Details uneinheitliche Ausgestaltung dieser Regelungen erschwert aber die Entwicklung einer gleichmäßigen und handlungssicheren Praxis. Und die prozeduralen Anforderungen begründen einen vielfach zeitintensiven Vorlauf bis zum Behandlungsbeginn. Infolgedessen kann es dazu kommen, dass Patienten, die eine Behandlung ablehnen, bei krankheitsbedingt hoher Aggressivität über längere Zeiträume einer mechanischen Sicherung oder – in Einzelfällen sogar über viele Monate – einer Unterbringung im Kriseninterventionsraum ausgesetzt sind, bis die schließlich als Ultima Ratio angeordnete Behandlung gegen ihren Willen die Aufhebung der Sicherungsmaßnahmen und eine einvernehmliche Fortsetzung der medikamentösen Behandlung wie der therapeutischen Arbeit möglich macht. Dass der Freiheitsentzug länger dauert, wenn die Symptomatik der Anlasserkrankung ohne Behandlung fortbesteht, bedarf keiner weiteren Erläuterung. Zu bemerken ist aber noch, dass es die Behandlungssituation für alle Patienten beeinträchtigt, wenn einzelne Patienten die Behandlung ablehnen und infolgedessen den Behandlungsalltag durch Krankheitssymptome belasten.

## Lösungsansätze

Das oberste Ziel muss sein, störungsbedingte Straftaten als Voraussetzung der Unterbringung psychisch kranker Menschen zum Schutz der Allgemeinheit zu verhüten, und zwar nicht erst durch Behandlung in einer Einrichtung des Maßregelvollzugs, sondern schon, bevor eine Unterbringung in einer solchen Einrichtung erforderlich wird.

Die geringe Rückfallquote schuldunfähiger Täter nach der Entlassung aus der Unterbringung spricht dafür, dass Menschen mit psychotischer Erkrankung von einem gut strukturierten stützenden Lebensumfeld, womöglich mit beschütztem Wohnen und Arbeiten, kontrollierten Weisungen und einer engen Anbindung an eine sozialarbeiterische und psychiatrische Nachsorge einschließlich Möglichkeiten der Krisenintervention sehr profitieren. Das rechtfertigt die Erwartung, dass vergleichbare Angebote und Maßnahmen auch kriminalpräventiv wirken, bevor es zu schwerwiegenden Straftaten und einer forensischen Unterbringung kommt.

Dies erfordert die rechtzeitige Bereitstellung ausreichender Angebote der Prävention und bedarfsorientierter Versorgungstrukturen. Häufig kommen Patienten zur Unterbringung, die im Vorfeld Behandlungen abgelehnt beziehungsweise nicht wahrgenommen haben. Hier ist eine frühzeitige engmaschige und niederschwellige Anbindung an allgemeinpsychiatrische Institutionen und die Einrichtung ausreichender psychiatrischer Wohnheime zielführend. Präventionsprojekte, die Patienten niederschwellig und aufsuchend betreuen, können bei kritischen Zuständen frühzeitig unterstützen und damit Straftaten und die daran gebundenen Unterbringungen verhindern.

Schon jetzt eröffnet das Psychisch-Kranken-Hilfe-Recht die Möglichkeit, die Vollziehung einer PsychK(H)G(Psychisch-Kranken-[Hilfe-]Gesetz)-Unterbringung unter Auflagen auszusetzen (§ 328 Gesetz über das Verfahren in Familiensachen und in den Angelegenheiten der freiwilligen Gerichtsbarkeit [FamFG]). Dieses Instrument sollte idealerweise konkretisiert und ausgebaut werden. Hilfreich könnten z. B. die Aufnahme eines Katalogs mit bestimmten Verhaltens‑, Behandlungs- und Kontaktauflagen in das Gesetz und die Ausweitung des zeitlichen Rahmens sein, für den solche Auflagen erteilt werden können. In bestimmten Fällen könnte auch die Möglichkeit einer (unter bestimmten Voraussetzungen auch ambulanten) medikamentösen Zwangsbehandlung indiziert und hilfreich sein.

Eine besondere Herausforderung stellt bei alledem die konkrete – und aus Verhältnismäßigkeitsgründen notwendig strikte – Eingrenzung der Voraussetzungen dar, unter denen diese erweiterten Eingriffsmöglichkeiten zur Anwendung gelangen dürfen. Gedacht werden könnte zum Beispiel daran, behutsam erweiterte Weisungs- und Eingriffsmöglichkeiten auf Fälle zu beschränken, in denen krankheitsbedingte Gefährdungssituationen schon einmal oder mehrfach in nicht ganz unerhebliche Schadensereignisse eingemündet waren. Auch dann muss es aber dabei bleiben, dass nicht die Behandlung zur Abwendung potenzieller Fremdgefährlichkeit oberstes Ziel bei der Sorge um Menschen mit psychischen Störungen ist. Vor allem muss es darum gehen, ihnen rechtzeitig und ausreichend Hilfe und Behandlung anzubieten und einen mit der unbehandelten Störung verbundenen Schaden abzuwenden. Hierzu zählen auch zu erwartende Progredienz der Beeinträchtigung, drohende Verwahrlosung und der Ausschluss von sozialer Teilhabe [9]. Dies betrifft also die schützenswerten Interessen der Betroffenen selbst, die auch eine Behandlung auf betreuungsrechtlicher Grundlage im Interesse der Betroffenen und der Fürsorge für die Patienten begründen können.

Die Unterbringung im psychiatrischen Maßregelvollzug ist ein schwerwiegender Eingriff und muss auf diejenigen Patienten beschränkt bleiben, die durch präventive Interventionen nicht erreicht werden können [7]. Sie muss so kurz wie möglich gestaltet werden und auf Reintegration zielen. Hierzu müssten die Rahmenbedingungen im psychiatrischen Maßregelvollzug bundesweit einheitlich und auskömmlich gestaltet werden. Zur Behandlung von Patienten mit Schizophrenie sind – nicht erst während der Unterbringung – forensisch geschulte Fachärzte für Psychiatrie und Psychotherapie unbedingt erforderlich [10]. Im Maßregelvollzug müssen die allzu häufig gefängnisähnlichen Strukturen auf Behandlungsangebote umgestellt und angepasst werden.

Um den Erfolg der Behandlung im Maßregelvollzug zu sichern, müssen auch nach der Unterbringung auskömmlich soziale Empfangsräume zur Verfügung stehen, die Patienten mit einem forensischen Hintergrund auch im allgemeinpsychiatrischen Versorgungssystem aufnehmen. Angesichts des steigenden Patientenaufkommens müssen mehr Heimplätze, beschützte und betreute Wohnmöglichkeiten, aber auch beschützte und betreute Arbeitsangebote geschaffen werden, um eine Reintegration der Betroffenen zu erleichtern. Die Schranken zwischen den Systemen des Maßregelvollzugs und dem nichtforensischen sozialen Versorgungssystem müssen hierzu durchlässiger und überbrückbar werden.

## Footnotes

1 Die Zahlen sind eingeschränkt zu vergleichen: Erst ab 2022 ist auch Bayern berücksichtigt. Außerdem kam es nach der Novellierung 2016 vorübergehend vermehrt zu Entlassungen aus Verhältnismäßigkeitsgründen [7].
